# Pharmacovigilance Bibliometrics: Visualizing Thematic Development in the Category of Pharmacology and Pharmacy in Web of Science

**DOI:** 10.3389/fphar.2021.731757

**Published:** 2021-10-04

**Authors:** Li Wang, Wanyu Feng, Jingli Duan, Jun Liang

**Affiliations:** ^1^ Department of Pharmacy, Peking University International Hospital, Beijing, China; ^2^ Department of Pharmacy, Peking University People’s Hospital, Beijing, China; ^3^ Department of Oncology, Peking University International Hospital, Beijing, China

**Keywords:** pharmacovigilance, bibliometrics, visualization, pharmacology and pharmacy, pharmacoepidemiolgy

## Abstract

**Introduction:** Pharmacovigilance studies include monitoring and preventing the occurrence of new, rare, or serious adverse drug reactions, making it possible to discover new safety issues without delay. Bibliometrics could assist scholars to analyze the development of pharmacovigilance.

**Methods:** The MeSH terms of both pharmacovigilance and “adverse drug reaction reporting system” were retrieved in the Science Citation Index Expanded. The articles from 1974 to July 2021 in the pharmacology and pharmacy category were recruited. The citation reports including the publication numbers, *h*-index, and sum and average cited times in terms of annuals, countries, organizations, authors and journals were tabulated. The coauthorship relations in the analysis units of countries, organizations, and authors; the top 10 burst references; the document citation network; and the author’s keywords co-occurrence overlay map were visualized by bibliometric software including the website (https://bibliometric.com/), VOSviewer, CiteSpace, and CitNetExplorer.

**Results:** From 1974 to the present, the most high-yield publication year, country, institute, author, and journal were 2020 (*n* = 222), France (*n* = 522), Netherlands Pharmacovigilance Centre Lareb (*n* = 82), Jean–Louis Montastruc (*n* = 125), *Drug Safety* (*n* = 384), respectively, in all 2,128 articles. Similarly, the United States, Institut National de la Sante et de la Recherche Medicale, and Jean–Louis Montastruc had the most coauthorship strength at the macrolevel (global), mesolevel (local), and microlevel (individual). The topics of burst references covered are the development of methodology, issues of patients reporting and under-reporting, evaluation of methods and databases, assessment of causality, and perspectives in pharmacovigilance. Eight clusters were grouped in the document citation network. “Pharmacovigilance,” “adverse drug reactions,” “pharmacoepidemiology,” “drug safety,” and “signal detection” were the research priorities, while “drug-related side effects and adverse reactions,” “VigiBase,” “disproportionality analysis,” “social media,” “FAERS,” “chemotherapy,” “patient safety,” “reporting odds ratio,” and “preventability” might be the future research hotspots.

**Conclusion:** Positive synergies can be observed in this study by employing the multiple software tools which established the relationship between the units of analysis. The bibliometric analysis can organize the thematic development and guide the hotspots of pharmacovigilance in pharmacology and pharmacy.

## Highlights:

1) Bibliometrics could assist scholars to analyze the development of pharmacovigilance over the past 4 decades and navigate the accurate target journals in manuscript submitting.
2) The year of 2020, the country France, the institution Netherlands Pharmacovigilance Centre Lareb, the author Jean-Louis Montastruc, and the journal *Drug Safety* made tremendous contribution in studies of pharmacovigilance.
3) The development of algorithms in spontaneous reporting systems, the issues of patients reporting and under-reporting, the evaluation of methods and database, the assessment of causality, and perspectives are the priority aspects in pharmacovigilance.
4) Various preventions and social media were utilized to guarantee the safety of patients. These aspects might become the future research hotspots.

## Introduction

The World Health Organization (WHO) defines pharmacovigilance as “the science and activities relating to the detection, assessment, understanding, and prevention of adverse effects or any other possible drug-related problems” in 2002 (WHO, 2002). The Medical Subject Heading (MeSH) terms of the PubMed database, adverse drug reaction reporting systems (NCBI, 1992), and pharmacovigilance (NCBI, 2012) are in the branch of “product surveillance and postmarketing” and introduced into the database since 1992 and 2012, respectively. The MeSH of bibliometrics (NCBI, 1990) is “the use of statistical methods in the analysis of a body of literature to reveal the historical development of subject fields and patterns of authorship, publication, and use.”

To timely detect the novel, rare, and serious adverse drug reactions (ADRs), various pharmacovigilance activities, such as clinical trials in the premarketing phase, data mining of the spontaneous reporting systems (SRSs) at the postmarketing stage, intensive monitoring with specific prescriptions during a certain period of time, and epidemiological studies based on the database or specific settings, were undertaken (Härmark and van Grootheest, 2008).

In the Web of Science (WoS) database, the Science Citation Index Expanded™ (SCIE) indexes more than 53 million records and 1.18 billion cited references from 1900 till present. Using the citation activity as the primary indicator, a total of 275 of the world’s most impactful journals in pharmacology and pharmacy category are selected into Journal Citation Reports™ (JCR) in 2020.

A bibliometric analysis and self-explanatory visualization tools could be the effective methods to evaluate the thematic development of structural contents and to assist readers’ understanding intuitively (Cobo et al., 2011). Combining the quantitative construction of the SCIE database and the qualitative contribution of the bibliometrics, the influential publications, research topic evolution, as well as the prominent authors, institutions, countries, and journals, could be accessed comprehensively.

To date, no bibliometric literature on pharmacovigilance topic has been analyzed and visualized. This research attempts to delineate the intellectual connections within the dynamic changing of scientific knowledge in the field of drug safety assurance by taking advantages of both the citation database and the software tools.

## Methods

### Data Retrieving

The WoS belongs to the commercial provider, Thomson Reuters, and requires an access fee. The database was accessed through the Peking University Health Science Library. The retrieval criteria were set as topic—pharmacovigilance or “adverse drug reaction reporting system,” and refined by the WoS category—Pharmacology Pharmacy, *Indexes = SCIE, Timespan = All years*. The document type of ARTICLE was included, whereas other document types such as EDITORIAL MATERIAL, NOTE, MEETING ABSTRACT, LETTER, CORRECTION, CORRECTION ADDITION, REVIEW, PROCEEDINGS PAPER, NEWS ITEM, and MEETING SUMMARY were excluded. The documents marked with EARLY ACCESS were also excluded because of the missing of the specific publication year and date information. Full records and references, citation reports, and JCR information were obtained on July 29, 2021.

### Bibliometric Analysis

The trends of publications and sum of times cited were charted annually. The records, percentage, *h*-index, sum of times cited, and average citations per item in terms of countries, organizations, and authors were tabulated directly. The coauthorship relations in the analysis units of countries, organizations, and authors were mapped by the bibliometric website (https://bibliometric.com/) and VOSviewer_1.6.17 edition (van Eck and Waltman, 2010) software, respectively. The full counting method, which means each coauthorship has the same weight, was applied in counting the records. The obtained records, percentage, *h*-index, sum of times cited, average citations per item, and JCR profiles (the country of the publisher, impact factor of 2020, rank, and quartile) of journals were tabulated. The burst references were calculated by CiteSpace_5.8. R1 edition (Synnestvedt et al., 2005) software, with settings: 1) the time span from 1974 to 2021; 2) the time slicing at 1 per year; and 3) the selection on top 10% of most cited in each slice (Egghe, 2006). The document citation network was composited by CitNetExplorer_1.0.0 edition (van Eck and Waltman, 2014) software with clustering parameters resolution of 1.00 and minimum cluster size of 30 articles. The preprocessing of duplicate detection and replacement (plural and singular, abbreviation, and full name) was applied to quality assurance. The author’s keywords co-occurrence overlay map was implemented by VOSviewer by setting the minimum occurrences of a keyword to 10 times.

Since all data were obtained from a public-available database and these software tools were free to use, this study does not require an ethical approval.

## Results

### Publication Outlines

**FIGURE 1:**
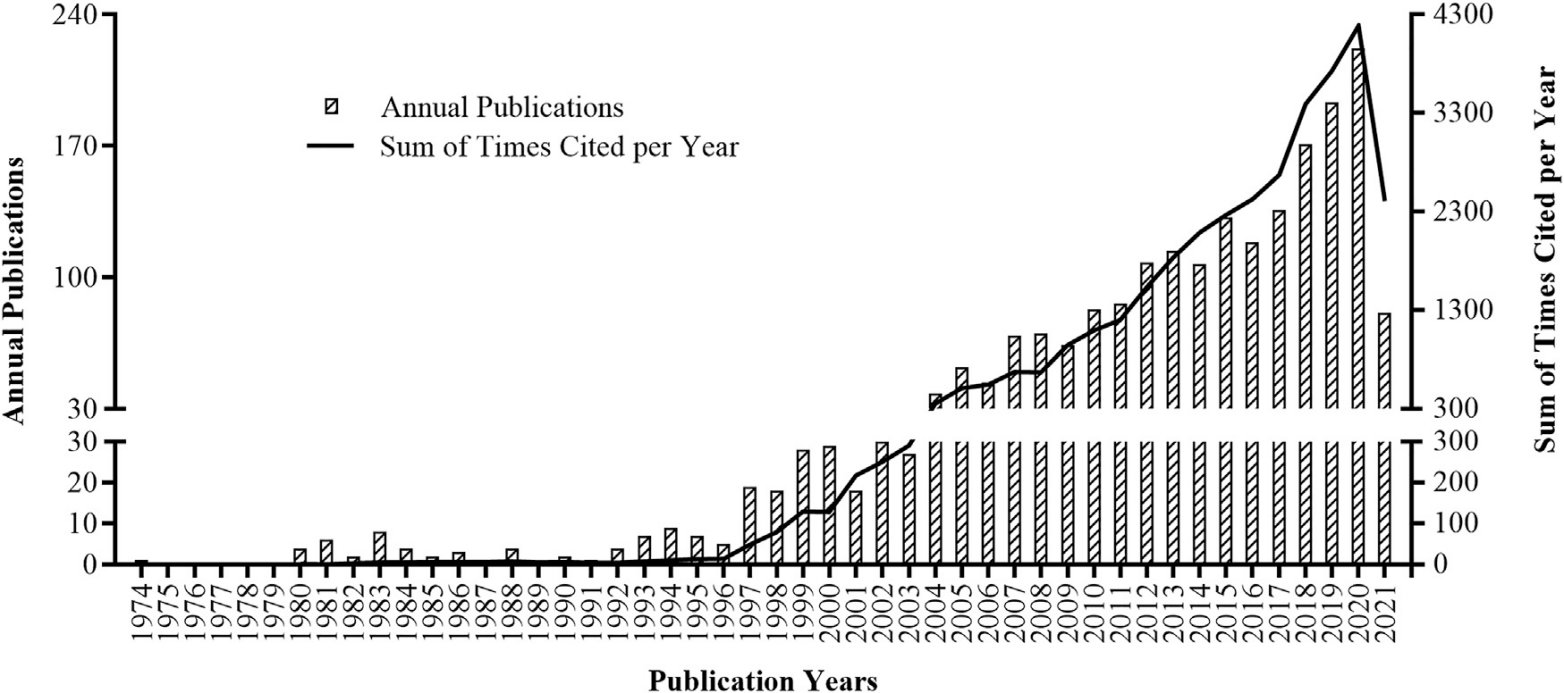
Annual publications (column chart) and sum of times cited per year (curve line) on pharmacovigilance from 1974 to 2021.

A total of 2,128 articles were retrieved in the SCIE from 1974 till present, with a sum of 33,791 times cited, average citations of 15.88 per item, and an *h*-index of 68. There are 1,959 articles written in English, and the rest were written in French (*n* = 158), German (*n* = 4), Portuguese (*n* = 4), Spanish (*n* = 4), and Japanese (*n* = 1). Figure 1 shows the annual publications and sum of times cited per year on pharmacovigilance. The first article was published in 1974, and the year with most publication (*n* = 222) was 2020. The citation started in 1980, and the year with most times cited was 2020 (*n* = 4,188).

### Contribution of Countries, Organizations, and Authors

There were 105 countries, 2,896 organizations, and 7,373 authors contributed to this topic, respectively. Table 1 summarizes the top five high-yield countries (France, the United States, the United Kingdom, the Netherlands, and Italy), organizations (Netherlands Pharmacovigilance Centre Lareb, Institut National de la Sante et de la Recherche Medicale [INSERM], Universite de Bordeaux, University of Groningen, and Uppsala Monitoring Centre), and authors (Jean–Louis Montastruc, Maryse Lapeyre–Mestre, Haleh Bagheri, Eugene P. van Puijenbroek, and Francois Montastruc), including the corresponding records, percentage, *h*-index, sum of times cited, and average citations per item.

**TABLE 1:** The top five high-yield countries, organizations, and authors on pharmacovigilance from 1974 to 2021.

Category	Rank	Items	Records (%)	*h*-index	Sum of times cited	Average citations per item
Country	1	France	522 (24.53)	40	7,529	14.42
	2	United States	365 (17.15)	39	6,260	17.15
	3	United Kingdom	300 (13.35)	41	7,029	23.43
	4	Netherlands	227 (10.67)	34	4,917	21.66
	5	Italy	170 (7.99)	28	2,905	17.09
Organization	1	Netherlands Pharmacovigilance Centre Lareb, the Netherlands	82 (3.85)	21	1,247	15.21
	2	Institut National de la Sante et de la Recherche Medicale (INSERM), France	56 (2.63)	17	1,036	18.5
	2′	Universite de Bordeaux, France	56 (2.63)	16	818	14.61
	2″	University of Groningen, the Netherlands	56 (2.63)	18	962	17.18
	2″′	Uppsala Monitoring Centre, Sweden	56 (2.63)	23	2,167	38.7
Author	1	Jean–Louis Montastruc, INSERM and Universite de Toulouse	125 (5.87)	25	2018	16.14
	2	Maryse Lapeyre–Mestre, INSERM and Universite de Toulouse	62 (2.91)	24	1,386	22.35
	3	Haleh Bagheri, INSERM and Universite de Toulouse	57 (2.68)	18	783	13.74
	4	Eugene P. van Puijenbroek, Netherlands Pharmacovigilance Centre Lareb	42 (1.97)	15	1,198	28.52
	5	Francois Montastruc, Universite de Toulouse	39 (1.83)	15	437	11.21

**FIGURE 2:**
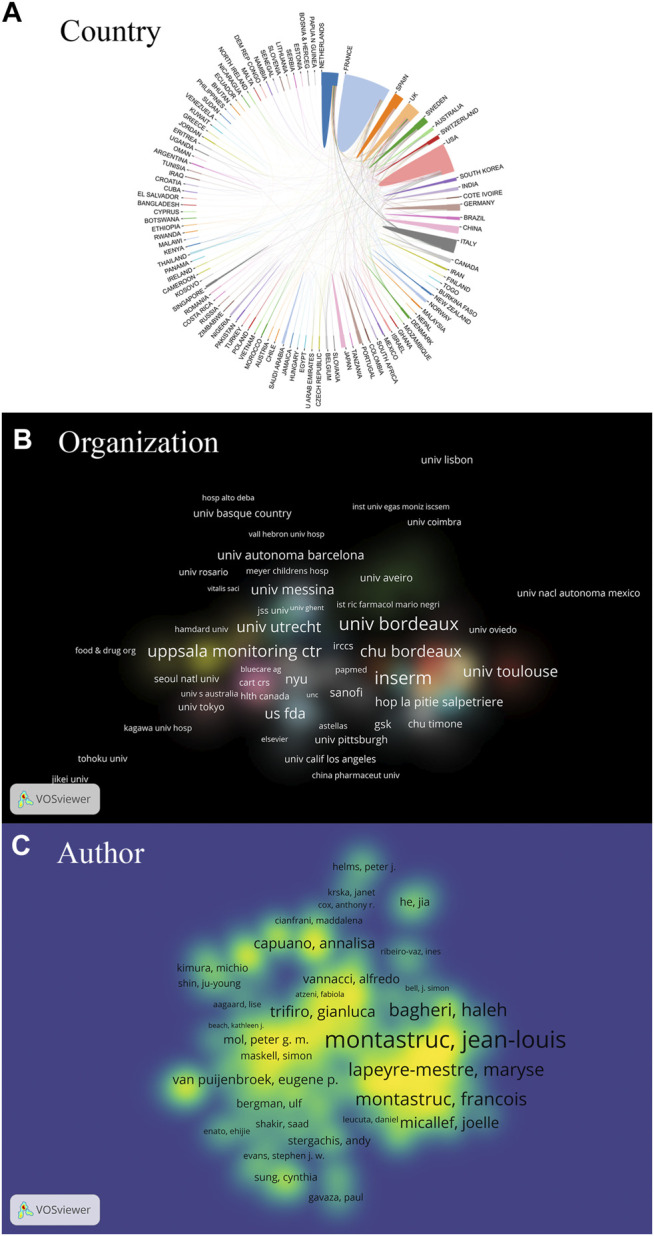
The coauthorship in terms of countries **(A)**, organizations **(B)** and authors **(C)** on pharmacovigilance from 1974 to 2021. The collaborative strength of countries is indicated by the circle area in Figure 2A. The coauthorship strength of organizations and authors is expressed by the label size in the brightened area of Figures 2B,C, respectively. The smaller distance between the two items reflects the stronger relation.

Figure 2 depicts the coauthorship strength at the macrolevel (global), mesolevel (local), and microlevel (individual). The strongest collaborative countries were the United States, the United Kingdom, Sweden, France, and Spain. The closest collaboration organizations were INSERM, Universite de Bordeaux, Uppsala Monitoring Centre, Centre Hospitalier Universitaire (CHU) de Bordeaux, and Universite de Toulouse. The authors with most coauthorship strength were Jean–Louis Montastruc, Maryse Lapeyre–Mestre, Haleh Bagheri, Joelle Micallef, and Francois Montastruc.

### Journals

There were 151 journals involved in this research. The top 10 high-yield journals and JCR profiles are tabulated in Table 1. *Drug Safety* occupied the most records (*n* = 384), the highest h‐index of 48, the highest sum of times cited (*n* = 9,674) and average citations per item (*n* = 25.2). *Frontiers in Pharmacology* had the highest impact factor of 5.81 in 2020. All the 10 journals came from developed countries from Europe, Oceania, and North America. These journals were distributed in four quartiles (each two in Q1, Q2, and Q4 and four in Q3) in the WoS category of Pharmacology and Pharmacy.

### Burst References

The top 10 high-burst references are gathered in Table 3. The reference “Use of Proportional Reporting Ratios (PRRs) for Signal Generation From Spontaneous Adverse Drug Reaction Reports” (Evans et al., 2001) had the earliest burst beginning year of 2003. One leading article entitled with “Use of Screening Algorithms and Computer Systems to Efficiently Signal Higher-Than-Expected Combinations of Drugs and Events in the US FDA’s Spontaneous Reports Database” (Szarfman et al., 2002) and two systematic reviews of “Under-Reporting of Adverse Drug reactions: A Systematic Review” (Hazell and Shakir, 2006) and “Determinants of Under-Reporting of Adverse Drug Reactions: A Systematic Review” (Lopez-Gonzalez et al., 2009) were all published on *Drug Safety*, the official journal of the International Society of Pharmacovigilance. The review of “Quantitative Signal Detection Using Spontaneous ADR Reporting” (Bate and Evans, 2009) owned the lowest burst strength of 11.21. In the past decade, three original research articles, “Experiences with Adverse Drug Reaction Reporting by Patients” (van Hunsel et al., 2012), “Performance of Pharmacovigilance Signal-Detection Algorithms for the FDA Adverse Event Reporting System” (Harpaz et al., 2013), and “vigiGrade: A Tool to Identify Well-Documented Individual Case Reports and Highlight Systematic Data Quality Issues” (Bergvall et al., 2014), had a long burst duration from 5 to 6 years. The most recent references were “Causality Assessment in Pharmacovigilance: The French Method and Its Successive Updates” (Miremont-Salamé et al., 2016) and “French Pharmacovigilance: Missions, Organization and Perspectives” (Vial, 2016), published on *Therapie*, while the former one got the highest burst strength of 14.82.

### Document Citation Network

**FIGURE 3:**
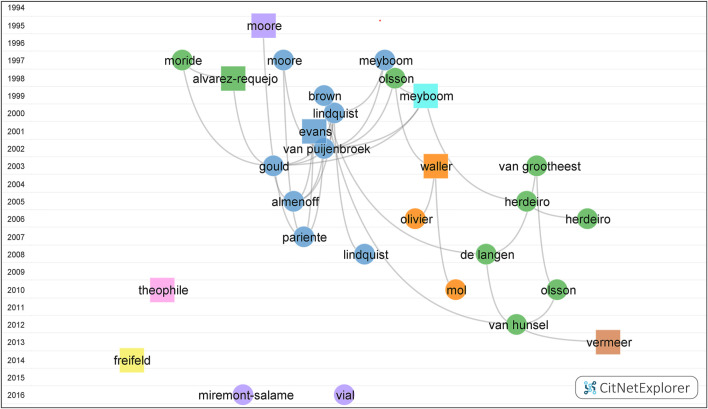
Documents Citation Network Visualization on pharmacovigilance from 1974 to 2021. The vertical axis coordinates indicate the publication year. Each dot/square indicates an article which is labeled with the last name of the first author. Each color marks a cluster. Group I to group VIII, in turn, were colored blue, green, purple, orange, yellow, brown, pink and cyan, respectively. The curved line annotates the citation relation. The square represents the publication with the highest citation score in each cluster.

Document citation network is visualized in Figure 3. Based on the citation link, eight groups were clustered, and the representatives of each group were depicted proportionally. Due to the minimum size requirement, 567 publications do not belong to any group.

The largest group (group I) (blue) contained 554 publications. Processing with the function of drill down, five more subgroups were clustered. The publication of Evans (Evans et al., 2001) got the top citation score (CS) of 166. The rest four articles were “The Medical Dictionary for Regulatory Activities (MedDRA)” (Brown et al., 1999), “VigiBase, the WHO Global ICSR Database System: Basic Facts” (Lindquist, 2008), “Principles of Signal Detection in Pharmacovigilance” (Meyboom et al., 1997), and “Impact of Safety Alerts on Measures of Disproportionality in Spontaneous Reporting Databases: The Notoriety Bias” (Pariente et al., 2007), which got the top CS of 94, 59, 53, and 51, respectively.

The second largest group (group II) (green) consisted of 489 articles. The articles in four subgroups were “Under-Reporting of Adverse Drug Reactions: Estimate Based on a Spontaneous Reporting Scheme and a Sentinel System” (Alvarez-Requejo et al., 1998), “Adverse Drug Reaction Reporting by Patients in the Netherlands: 3 Years of Experience” (de Langen et al., 2008), “Influence of Pharmacists’ Attitudes on Adverse Drug Reaction Reporting: A Case-Control Study in Portugal” (Herdeiro et al., 2006), and “Pharmacovigilance Activities in 55 Low- and Middle-Income Countries: A Questionnaire-Based Analysis” (Olsson et al., 2010), which got the top CS of 53, 42, 33, and 31, respectively.

Group III to Group VII, in turn, were colored purple (177 articles), orange (142 articles), yellow (54 articles), brown (51 articles), pink (50 articles), and cyan (44 articles), respectively. The articles with the highest CS in the remaining six groups in turn were Moore (CS = 37) (Moore et al., 1995), Waller (CS = 21) (Waller and Evans, 2003), Freifeld (CS = 16) (Freifeld et al., 2014), Vermeer (CS = 18) (Vermeer et al., 2013), Theophile (CS = 12) (Théophile et al., 2010), and Meyboom (CS = 18) (Meyboom et al., 1999).

### Keywords Co-Occurrence

**FIGURE 4:**
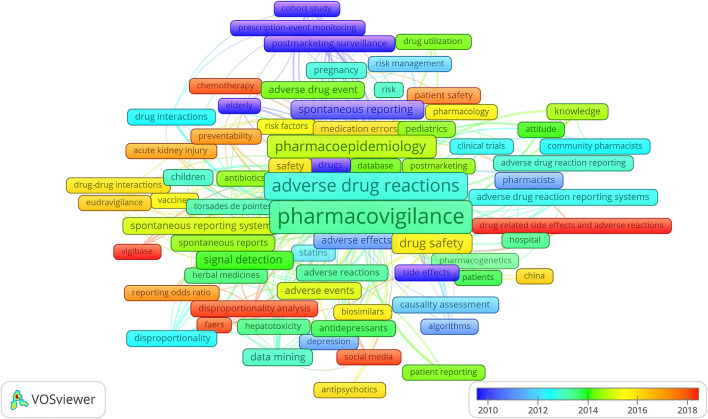
Keywords’ co-occurrence overlay mapping on pharmacovigilance from 1974 to 2021. Each frame indicates a keyword. The rainbow color marks the average publication year from violet (further year) to red (recent year) in the range of spectrum. The larger scale of a keyword is according to the higher frequency, while the closer distance between the two keywords represents the stronger co-occurrence.

The overlay visualization of the top 69 co-occurrence keywords is drawn in Figure 4. The highest occurrence keywords were “pharmacovigilance” (*n* = 906), “adverse drug reactions” (*n* = 485), “pharmacoepidemiology” (*n* = 215), “drug safety” (*n* = 125), and “signal detection” (*n* = 79). The most recent keywords were “drug-related side effects and adverse reactions” and “VigiBase” (the average year of 2018), “disproportionality analysis,” “social media,” “FAERS,” “chemotherapy,” “patient safety,” “reporting odds ratio,” and “preventability” (the average year of 2017).

## Discussion

Bibliometrics, based on the quantitative and intuitive properties, can objectively analyze the hotspots and trends in specific research fields. A comparative study was performed among the popular and reliable databases, namely, PubMed, Scopus, and WoS (Falagas et al., 2008). PubMed is accessed for free and considered as an optimal tool in biomedical research. However, it does not provide citation analysis. Scopus and WoS have the ability to analyze the citations. The WoS covers the publications and citations dating back to 1900, while Scopus indexes literature studies since 1966 and the citation analysis for articles published after 1996. Besides, the WoS annually releases the journal impact factor, a tool for evaluating the importance and influence of specific publications. At last, the WoS database was selected to implement our research as both the burst references and the document citation network were based on up-to-date citation analysis. This study, for the first time, reports the scientific metric indicators, identifies the core contributors, visualizes the knowledge development, and assesses the citation relationship on the pharmacovigilance topic in the category of pharmacy and pharmacology in the WoS database.

### Analysis on Publication Outlines

The publication amount of a research topic can reflect the popularity of the whole year. Pharmacovigilance studies have developed in the past half-century. The annual articles before 1996 were initially published in single digits and exceeded 38 since 2004. From 2004 to 2020, there existed a rough linear rise for the outputs and an exponential growth for the citation times with the publication year, respectively. The future trend indeed looks promising and blossoming. The increasing application of new drugs or new indications of approved drugs by the authorities all over the world boosted pharmacovigilance research. The WoS database also indexes the funding agencies. The top five national or industrial agencies, including the European Commission, United States Department of Health Human Services, National Institutes of Health in the United States, GlaxoSmithKline, and Pfizer, powered the outputs since the initially launched funding in 2003 (funding data were not shown).

### Analysis on Contribution of Countries, Organizations, and Authors

Tracking the high yield and strong co-operation under the analysis units of countries, organizations, and authors is commonly performed to obtain the social structure of a research topic. Scholars can seek further collaboration on the interesting topics. As a combined analysis of Table 1 and Figure 2, the authors’ scientific achievements are relevant to the affiliations in terms of organizations and countries. The top three high-yield authors, Jean–Louis Montastruc, Maryse Lapeyre-Mestre, and Haleh Bagheri, were also the top three strong collaborative individuals, who were from the INSERM, which was ranked in the top five organizations with both high amounts and strong co-operations. Furthermore, the contributions of those 158 French-written articles should be noticeable. All efforts are the total contributions of France among countries all over the world. Of course, a bunch of national/regional studies productivity has been established within the scope of national/regional funding. The quantitative indicators consisted of *h*-index, sum of times cited, and average citations per item, which change dynamically with the research evolution and can point out the significant items.

These developed countries seem to have superior conditions for pharmacovigilance research based on the drug monitoring centers, including the WHO Uppsala Drug Monitoring Centre in Sweden (Sweden ranked seventh in terms of publication records; data were not shown), Netherlands Pharmacovigilance Centre Lareb in the Netherlands, Italian Pharmacovigilance Adverse Event Spontaneous Reporting System in Italy, the regulatory authorities, including the Medicines and Healthcare products Regulatory Agency in the United Kingdom, Food and Drug Administration (FDA) in the United States, and the academia/national research institutes, including the INSERM in France etc*.*

### Analysis on Journals

The identification of the dominant journals in a specific topic can assist scholars’ construction of scientific achievement. JCR profiles are journal-specific metrics involving the impact factor, the rank, and quartile in the category. Active journals not only provide prominent information but also prompt accurate submitting navigation. A high burst signal of a reference implies a high intensity of research interest along with the time interval (Kleinberg, 2003). As shown in Tables 2 and 3, it is worth noting that nine out of the top 10 references with the strongest citation bursts were published on the highest yield journals, *Drug Safety*, *Pharmacoepidemiology and Drug Safety*, and *Therapie*. Focusing on high-yield and burst core journals can assist researchers to access not only the most authoritative knowledge framework but also the orientation of manuscript submitting. The publishers of four journals belong to the United States, while the rest are all from the developed countries, which make the high productivity sense.

**TABLE 2:** The top 10 high-yield journals on pharmacovigilance from 1974 to 2021.

Rank	Journals	Records (%)	*h*-index	Sum of times cited	Average citations per item	Country of the publisher	Impact factor 2020	Rank	Quartile
1	*Drug Safety*	384 (18.05)	48	9,674	25.2	New Zealand	5.606	46/275	Q1
2	*Pharmacoepidemiology and Drug Safety*	272 (12.78)	37	5,841	21.47	United States	2.89	170/275	Q3
3	*Therapie*	229 (10.76)	18	1,550	6.77	Netherlands	2.07	216/275	Q4
4	*British Journal of Clinical Pharmacology*	134 (6.30)	33	3,271	24.41	United States	4.335	90/275	Q2
5	*European Journal of Clinical Pharmacology*	133 (6.25)	28	2,547	19.15	Germany	2.953	167/275	Q3
6	*Frontiers in Pharmacology*	61 (2.87)	11	384	6.3	Switzerland	5.81	39/275	Q1
7	*International Journal of Clinical Pharmacy*	48 (2.26)	11	316	6.58	Netherlands	2.054	217/275	Q4
8	*Fundamental Clinical Pharmacology*	45 (2.12)	12	500	11.11	United States	2.748	176/275	Q3
9	*Expert Opinion on Drug Safety*	41 (1.93)	6	143	3.49	England	4.25	93/275	Q2
10	*Journal of Clinical Pharmacy and Therapeutics*	41 (1.93)	12	408	9.95	United States	2.512	195/275	Q3

**TABLE 3:** Top 10 references with the strongest citation bursts on pharmacovigilance from 1974 to 2021.

References	Strength	Begin	End	2001–2021^a^
Evans et al. (2001), *PHARMACOEPIDEM DR S*, V10, P483	14.58	2003	2006	▂▂▃▃▃▃▂▂▂▂▂▂▂▂▂▂▂▂▂▂▂
Szarfman et al. (2002), *DRUG SAFETY*, V25, P381	12.41	2004	2007	▂▂▂▃▃▃▃▂▂▂▂▂▂▂▂▂▂▂▂▂▂
Hazell and Shakir (2006), *DRUG SAFETY*, V29, P385	12.32	2008	2011	▂▂▂▂▂▂▂▃▃▃▃▂▂▂▂▂▂▂▂▂▂
Lopez-Gonzalez et al. (2009), *DRUG SAFETY*, V32, P19	12.72	2011	2014	▂▂▂▂▂▂▂▂▂▂▃▃▃▃▂▂▂▂▂▂▂
Bate and Evans (2009), *PHARMACOEPIDEM DR S*, V18, P427	11.21	2012	2014	▂▂▂▂▂▂▂▂▂▂▂▃▃▃▂▂▂▂▂▂▂
van Hunsel et al. (2012), *DRUG SAFETY*, V35, P45	11.54	2013	2017	▂▂▂▂▂▂▂▂▂▂▂▂▃▃▃▃▃▂▂▂▂
Harpaz et al. (2013), *CLIN PHARMACOL THER*, V93, P539	13.83	2013	2018	▂▂▂▂▂▂▂▂▂▂▂▂▃▃▃▃▃▃▂▂▂
Bergvall et al. (2014), *DRUG SAFETY*, V37, P65	11.95	2015	2019	▂▂▂▂▂▂▂▂▂▂▂▂▂▂▃▃▃▃▃▂▂
Miremont-Salamé et al. (2016), *THERAPIE*, V71, P179	15.01	2016	2021	▂▂▂▂▂▂▂▂▂▂▂▂▂▂▂▃▃▃▃▃▃
Vial (2016), *THERAPIE*, V71, P143	14.04	2017	2021	▂▂▂▂▂▂▂▂▂▂▂▂▂▂▂▃▃▃▃▃▃

a 2021 means from January to July of 2021.

### Analysis on Burst References

Aimed on discovering the core research, the document cocitation analysis can settle down the inherent relationship in the knowledge structure, while the burst detection analysis (Kleinberg, 2003) extracts the high intensity references over finite durations through CiteSpace software. In addition, recent publications might not have sufficient citations mainly because citation analysis is time-dependent.

The chronological topic evolution of the burst references covers these aspects: methodology development (Evans et al., 2001; Szarfman et al., 2002; Bate and Evans, 2009), patients reporting and under-reporting issues (Hazell and Shakir, 2006; Lopez-Gonzalez et al., 2009; van Hunsel et al., 2012), methods and database evaluation (Bate and Evans, 2009; Bergvall et al., 2014), causality assessment (Miremont-Salamé et al., 2016), and perspectives (Vial, 2016) in pharmacovigilance.

Traditional pharmacovigilance methods involve literature searching, case-by-case analysis, frequency counts, and the calculation of reporting rates (Almenoff et al., 2005). Aimed to timely discover novel ADRs, the data mining algorithms are developed, assessed, and utilized by health-associated communities and researchers. In the context of signal detection, data mining algorithms include the frequentist (PRR; reporting odds ratios, ROR), the Bayesian (Bayesian confidence propagation neural network; multi-item gamma-Poisson shrinker, MGPS), and multivariate modeling techniques such as logistic regression (Hauben et al., 2005; Harpaz et al., 2013). These activities ensure that early signs of previously unknown medicine-related safety problems are identified as rapidly as possible. However, there are still concerns about the lack of systematic, objective validation of the methods in both traditional and computer-enhanced data mining methods. At present, there are no standards or guidelines for the methods of data mining in routine pharmacovigilance. Disproportionality analysis could assist scholars to discover drug-event associations based on SRSs. Evans *et al.* developed PRR in the United Kingdom Yellow Card database, in which 15 newly marketed drugs with the highest levels of ADR reporting from 1996 to 1998 were retrospectively investigated (Evans et al., 2001). The results show the proportions of recognized ADR, events considered to be related to the underlying disease, and events requiring further evaluation were 70, 13, and 17%, respectively. Szarfman *et al.* from the Office of Biostatistics, the Center for Drug Evaluation and Research, FDA, United States, summarized the technical aspects of the MGPS algorithm (Szarfman et al., 2002). The early identification process of ADRs in SRSs could assist to minimize the potential drug toxicity risks of patient safety. Various data mining methods were utilized in the pharmacovigilance arena. Bate *et al.* insisted that quantitative signal detection methods in large spontaneous reports database could assist cost-effectively in possible new ADRs (Bate and Evans, 2009).

As one of the main shortcomings of the SRSs, the under-reporting of ADRs was summarized in two review articles (Hazell and Shakir, 2006; Lopez-Gonzalez et al., 2009). Hazell *et al.* estimated the extent of 6–100% (median 94%) under-reporting ADR rates to SRSs through 37 studies from 12 countries under different settings (hospital setting or general practice), database (hospital admission/discharge, insurance, or background), and drugs (all drugs or specific drugs) (Hazell and Shakir, 2006). Lopez-Gonzalez *et al.* systematically reviewed 45 publications on the topic of ADR under-reporting determinants and displayed the knowledge and attitudes of health professionals would be related with high reporting rates, compared with personal and professional factors (Lopez-Gonzalez et al., 2009). The reporting of ADRs from patients is conductive for pharmacovigilance, which was widely recognized by the 11 countries (Australia, Canada, Denmark, the Netherlands, New Zealand, Norway, Malaysia, Philippines, Sweden, the United Kingdom, and United States) based on telephone interviews, email discussion, and field visits (van Hunsel et al., 2012).

Evaluation of the signal detection algorithms could assist to identify the reliable methods, while the improvement of the SRSs should assure the original quality of structural information. The application of computerized tools offers the opportunity to analyze data in a consistent and timely manner. Harpaz *et al.* firstly carried out a systematic examination of the sensitivity–specificity trade-off to distinguish the properties of disproportionality analysis and multivariate modeling. Logistic regression and MGPS are superior to other algorithms in each technique (Harpaz et al., 2013). Bergvall and colleagues proposed the vigiGrade tool to measure the completeness and identify the predictors of the overall well-documented individual case reports in VigiBase (Bergvall et al., 2014).

### Analysis on Documents Citation Network and Keywords Co-Occurrence

The visualization of documents citation network can sort the intellectual base thematically in which clustering algorithms are widely used. Based on the algorithmic historiography peculiarity, the citation network of bibliometrics took the advantages of evaluating articles through citation relations. Therefore, all retrieved articles from the database can be analyzed objectively, compared with the traditional reviews which were influenced by scholars’ own opinions.

The coword analysis by using similarity measures focuses on the conceptual structure evolution of a scientific field. Keywords co-occurrence overlay maps can provide both fundamental topics (the scale size is positively correlated with the occurrence frequency in the visualization) and recent hotspots (the average publication year is positively correlated with warm and cold colors).

In group I of Figure 3, five core aspects on pharmacovigilance were discussed chronologically:

1) Principles: Meyboom *et al.* reported seven basic principles of signal detection—association strength, data consistency, exposure–response relationship, biological plausibility, experimental findings, possible analogies, and the nature and quality of the data (Meyboom et al., 1997).
2) Terminology: Brown *et al.* delineated the MedDRA terminology which was widely used in the pre- and postmarketing phase of the medicine (Brown et al., 1999). In Figure 4, the preferred terms “torsades de pointes” and “hepatotoxicity” occurred frequently. On behalf of the Pharmaceutical Research and Manufacturers of America-FDA Collaborative Working Group on Safety Evaluation Tools, Almenoff *et al.* summarized the definitions of pharmacovigilance terms and emphasized the importance of terminology (Almenoff et al., 2005).
3) Methodology: in order to better identify, analyze, and characterize the signals from pharmacovigilance, the methodology evolved in the last 50 years. Evans *et al.* developed the PRR (Evans et al., 2001), and Van Puijenbroek *et al.* examined the concordance level of the five different estimates including the ROR, PRR, Yule’s Q, Poisson’s probability, and Chi-square test to the information component measure used by the WHO Uppsala Monitoring Centre (van Puijenbroek et al., 2002). At the beginning of the pharmacovigilance study, methodologies such as “prescription-event monitoring” and “cohort study” (colored purple in Figure 4) were widely utilized in postmarketing surveillance. “Signal detection” which occurred 74 times in total and “disproportionality analysis” with orange color which indexed the average publication year around 2017 in Figure 4 demonstrated the prominent and promising characteristics of these related topics.
4) Database: the WHO operates the global individual case safety report database, VigiBase (Lindquist, 2008), the European Medicines Agency (EMA) maintains the EudraVigilance Data Analysis System, and the FDA runs the Adverse Event Reporting System (FAERS) (Dal Pan and Arlett, 2015). As shown in Figure 4, “VigiBase,” “FAERS,” and “EudraVigilance” were colored red, orange, and red, respectively. These three large regulatory databases are holding the tasks of collecting and analyzing ADR reports; maintaining drug safety surveillance systems; developing the theory, methodology, practice, and research of pharmacovigilance; and communicating the safety message throughout the world. Aimed to quantify the redundancy among the three databases, a comparative study which was applying the signals of disproportionate reporting indicated the characteristics of regulatory expectations, operating performance, and procedural complexity were largely similar (Vogel et al., 2020). Focused on detecting the unexpected or unknown new side effects, the SRSs collect the suspected ADRs when the products are marketed. Having taken the advantage of entire patient population and drug range, the SRSs became a cost-effective method in the pharmacovigilance field. As the development of SRSs, the disproportionality analysis has sprung up and is expected to become a research hotspot in the future.
5) Reporting bias: Pariente *et al.* reported the bias based on French SRS database through a study using the ROR and its 95% confidence interval, in which the disproportionality increased after a safety alert (Pariente et al., 2007).

In group II of Figure 3, four dominating points on pharmacovigilance were explored:

1) Under-reporting: Moride *et al.* quantitatively assessed the general practice in France and found greater efficacy in serious and unlabeled effects (Moride et al., 1997), and Alvarez-requejo *et al.* confirmed and quantified the extent of under-reporting in general practice in Spain and assessed the influence factors (Alvarez-Requejo et al., 1998).
2) Attitude: the studies of Herdeiro *et al*. indicated the attitude of healthcare providers including pharmacists (Herdeiro et al., 2006) and physicians (Herdeiro et al., 2005) were strongly associated with the under-reporting ADRs.
3) Country: Olsson *et al*. identified barriers to promote pharmacovigilance in low- and middle-income countries (Olsson et al., 2010). “China” with the yolk color in Figure 4, on behalf of the largest developing country, began to contribute on pharmacovigilance research.
4) Patient reporting: de Langen *et al.* confirmed that patient reporting in SRSs is feasible and contributes significantly to a reliable pharmacovigilance in a 3-year period study based on the Netherlands Pharmacovigilance Centre Lareb (de Langen et al., 2008). Van Hunsel *et al.* comprehensively reviewed the importance of patients reporting of ADRs in 11 countries (van Hunsel et al., 2012).

In group III, as a major player in European and world pharmacovigilance, France contributed tremendously. The French pharmacovigilance system was created at the end of the 1970s. Thirty-one regional pharmacovigilance centers across the country are collecting ADRs from the public, such as patients and health professionals, while the routine departments of pharmacovigilance in industry are summarizing safety reports. Data from these two approaches are evaluated and processed at the decisional authority, the French Agency for the Safety and Health Products. This decentralized organization has proved to be efficient in detecting signals. The improved communication and transparency through websites and IT networks have powered the performance among France and the EMA (Vial, 2016). Miremont–Salame’s article reviewed the causality assessment algorithm, which consisted of three chronological scores, four semiological scores, and one bibliographic score in France, where the method was initially published in 1978 and updated twice in 1985 and 2011 (Miremont-Salamé et al., 2016). Vial *et al.* described the missions of the French pharmacovigilance system, involving the French drug agency, regional centers of pharmacovigilance, health professionals, pharmaceutical companies, patients, and their associations (Vial, 2016).

Four burst articles were also marked in groups I (Evans et al., 2001), II (van Hunsel et al., 2012), and III (Miremont-Salamé et al., 2016; Vial, 2016) of the citation network. The topics of quantitative signal detection, the patient reporting, and the causality are the momentous aspects of pharmacovigilance.

In group IV of Figure 3, authorities provoked the scientific conductive model such as the risk management plans should be developed for the safety knowledge (Waller and Evans, 2003). Keyword “risk management” (blue) was also a high frequent topic in Figure 4. A branch of researchers raised the concerns on medical products’ safety surveillance monitoring as the increasing social media (Freifeld et al., 2014) in group V. Keyword “social media” was colored red in Figure 4, which implied the current hotspot and a rising future trend. The safety profile of biopharmaceuticals (Vermeer et al., 2013) in group VI attracted the attention of the EU and FDA in recent years and studied in different aspects, including various databases, the accuracy of product names, or drug-related reactions. Causality assessment can be described as the estimation of the causal relationship between a drug and an adverse event. As the main theme of group VII, Theophile *et al.* compared three methods of ADR causality assessment, including consensual expert judgment, algorithmic, and probabilistic approaches, based on the Bordeaux Pharmacovigilance Centre in France. The first method showed the satisfactory assessment (Théophile et al., 2010).

The targeted population, such as gender differences (Montastruc et al., 2002) and elderly and pediatric patients (Horen et al., 2002), is discussed in Figure 4. The scholars had focused on groups transferring from the “elderly” (violet), to “pregnancy” and “children” (cyan), to “pediatrics” (green) according to the average publication year of the keywords. The specific medicine categories of study changed from “statins” (blue), to “herbal medicine” (Barnes, 2003), “antidepressants” and “antibiotics” (green), to “vaccines” (yellow), to “biosimilars” and “antipsychotics” (orange), to chemotherapy (red). The distance between two items in Figure 4 could reflect the correlation. “Hepatotoxicity” was close to “herbal medicine” which was consistent with the findings of high drug-induced liver injury occurrence under the herbal regimens (Cao et al., 2021). In addition, there are several other categories in the WoS database that focus pharmacovigilance, involving vaccine safety and immunization in immunology, schizophrenia, and antipsychotics in psychiatry, epilepsy and antiepileptic drugs in clinical neurology, malaria and artemether, HIV and antiretroviral therapy in infectious diseases, and immune checkpoint therapy in oncology. All the pharmacovigilance findings in these therapeutic fields alert the agency on the potential threats to the public health. Agency experts then identify the need for preventive actions, such as changes in product labeling information and, rarely, re-evaluation of an approval decision.

### Limitations

Aimed to guarantee a high-quality bibliometric analysis, we only implemented this work under the restriction of the pharmacology and pharmacy category in the WoS database. A considerable amount of research was missed because they were published in non-SCI journals or grouped into other categories. Besides, some non-English literature stuides would be ignored as the WoS database was originated from the United States. The WHO Programme for International Drug Monitoring was started in 1968, but the glossary of “pharmacovigilance” was defined since 2002. And the same goes for the MeSH term “pharmacovigilance,” which was introduced since 2012 in the PubMed database. There might be a lack of consistency of terminology in the literature studies at the beginning years, which could lead to a number of overlooked studies. In addition, bibliometrics is lacking the ability to assess the quality of individual studies as the indicator of citation is time-dependent, which means recent articles may not have sufficient citations than the previous articles mainly because of the publication date.

## Conclusions

Based on the studies of pharmacovigilance over the past 4 decades, we assessed the literature information regarding different years, countries, affiliations, authors, and journals and analyzed the thematic development and the future research hotspots. To obtain the validity and utility framework of this research field, integration of both the quantitative and the qualitative comparison of the characteristics is considered highly reliable for the scholars. To conclude, our research observes the raising concern on pharmacovigilance in recent years. *Drug Safety*, *Pharmacoepidemiology and Drug Safety*, and *Therapie* are the most influential journals. The priority topics include the SRSs and the computerized algorithms, such as VigiBase, FAERS, and disproportionality analysis. With the aim of guaranteed patient safety, various preventions and social media are becoming the future hotspots in pharmacovigilance research.

## Data Availability

The original contributions presented in the study are included in the article/supplementary material. Further inquiries can be directed to the corresponding authors.

## Abbreviations

WHO, World Health Organization; MeSH, Medical Subject Headings; ADR, adverse drug reaction; SRS, spontaneous reporting system; WoS, Web of Science; SCIE, Science Citation Index Expanded; JCR, Journal Citation Reports; INSERM, Institut National de la Sante et de la Recherche Medicale; CHU, Centre Hospitalier Universitaire; PRR, proportional reporting ratio; CS, citation score; MedDRA, Medical Dictionary for Regulatory Activities; FDA, Food and Drug Administration; ROR, reporting odds ratios; MGPS, multi-item gamma-Poisson shrinker; EMA, European Medicines Agency; FAERS, FDA Adverse Event Reporting System.
